# Effects of Charge Compensation on Colossal Permittivity and Electrical Properties of Grain Boundary of CaCu_3_Ti_4_O_12_ Ceramics Substituted by Al^3+^ and Ta^5+^/Nb^5+^

**DOI:** 10.3390/molecules26113294

**Published:** 2021-05-30

**Authors:** Jakkree Boonlakhorn, Jedsada Manyam, Pornjuk Srepusharawoot, Sriprajak Krongsuk, Prasit Thongbai

**Affiliations:** 1 Giant Dielectric and Computational Design Research Group (GD–CDR), Department of Physics, Faculty of Science, Khon Kaen University, Khon Kaen 40002, Thailand; jakkree_boonlakhorn@hotmail.com (J.B.); spornj@kku.ac.th (P.S.); srikro@kku.ac.th (S.K.); 2 Institute of Nanomaterials Research and Innovation for Energy (IN–RIE), NANOTEC–KKU RNN on Nanomaterials Research and Innovation for Energy, Khon Kaen University, Khon Kaen 40002, Thailand; 3 National Nanotechnology Center (NANOTEC), National Science and Technology Development Agency (NSTDA), Pathum Thani 12120, Thailand; jedsada@nanotec.or.th

**Keywords:** DFT calculation, giant dielectric permittivity, impedance spectroscopy, nonlinear current-voltage characteristics, DC bias

## Abstract

The effects of charge compensation on dielectric and electrical properties of CaCu_3_Ti_4-*x*_(Al_1/2_Ta_1/4_Nb_1/4_)*_x_*O_12_ ceramics (*x* = 0−0.05) prepared by a solid-state reaction method were studied based on the configuration of defect dipoles. A single phase of CaCu_3_Ti_4_O_12_ was observed in all ceramics with a slight change in lattice parameters. The mean grain size of CaCu_3_Ti_4-*x*_(Al_1/2_Ta_1/4_Nb_1/4_)*_x_*O_12_ ceramics was slightly smaller than that of the undoped ceramic. The dielectric loss tangent can be reduced by a factor of 13 (tanδ ~0.017), while the dielectric permittivity was higher than 10^4^ over a wide frequency range. Impedance spectroscopy showed that the significant decrease in tanδ was attributed to the highly increased resistance of the grain boundary by two orders of magnitude. The DFT calculation showed that the preferential sites of Al and Nb/Ta were closed together in the Ti sites, forming self-charge compensation, and resulting in the enhanced potential barrier height at the grain boundary. Therefore, the improved dielectric properties of CaCu_3_Ti_4-*x*_(Al_1/2_Ta_1/4_Nb_1/4_)*_x_*O_12_ ceramics associated with the enhanced electrical properties of grain boundaries. In addition, the non-Ohmic properties were also improved. Characterization of the grain boundaries under a DC bias showed the reduction of potential barrier height at the grain boundary. The overall results indicated that the origin of the colossal dielectric properties was caused by the internal barrier layer capacitor structure, in which the Schottky barriers at the grain boundaries were formed.

## 1. Introduction

Colossal dielectric permittivity of ceramic oxides with very large dielectric constant (*ε*′ > 10^4^) has been extensively studied for use in electronics applications, especially for capacitive-based devices such as ceramic capacitors [1,2,3,4,5,6,7,8,9,10,11]. Since the colossal dielectric permittivity of CaCu_3_Ti_4_O_12_ (CCTO) ceramics was reported by Subramanian, et al. [12], many simple and complex oxides such as NiO, ZnO, TiO_2_, SnO_2_, BiFeO_3_, and CCTO-based ceramics, have been investigated [1,2,3,13,14,15,16,17,18,19,20,21,22,23,24]. However, these ceramic oxides have limitations for application in electronic devices. One of the most serious problem is a large value of the dissipation factor (tan*δ*) [1,2,15,25,26].

In addition, the primary causes of the colossal permittivity and nonlinear electrical response of CCTO ceramics have also been investigated due to the arguments of various models [16,27,28,29]. An internal barrier layer capacitor (IBLC) model is primarily accepted to be the origin of colossal permittivity in CCTO due to its exceptionally heterogeneous microstructure, consisting of a highly resistive layer of the grain boundaries and more conductive core inside of the grains [16,27]. However, in the case of substitution metal ions, intrinsic properties corresponding to the electronic structure and defect structures have a strong influence on the dielectric properties [28]. Therefore, an investigation on both extrinsic related-microstructure and intrinsic properties is essential.

Recently, improved colossal dielectric permittivity of these oxides can be observed in single and codoped CCTO ceramics [1,2,15,17,30,31,32,33,34,35,36]. Considering the heteroatomic substitution method, colossal permittivity with low tan*δ* was reported in (A^5+^, B^3+^) codoped TiO_2_ ceramics such as (Nb^5+^, In^3+^) [1], (Nb^5+^, Al^3+^) [35], (Nb^5+^, Ga^3+^) [34], and (Nb^2+^, Sc^3+^) [36] codoped TiO_2_ ceramics. Song, et al. [15] reported a high *ε*′ > 10^3^ and low tan*δ* ~0.015 in Al*_x_*Nb_0.05_Sn_0.95-*x*_O_2_.

By using the codoped concept, improved dielectric properties with low tanδ ~0.045 to 0.058 and high *ε*′ ~2.9 to 4.1 × 10^4^ were achieved in CaCu_3_Ti_4-*x*_(Nb_1/2_Al_1/2_)*_x_*O_12_ ceramics [2]. The investigation in codoped CCTO ceramics is of greater interest than those of other oxides because CCTO polycrystalline ceramics can also exhibit non-Ohmic properties, which can be used in varistors devices. Self-charge compensation in CaCu_3_Ti_4-*x*_(Nb_1/2_Al_1/2_)*_x_*O_12_ ceramics was proposed to be the primary cause of the enhanced colossal permittivity. However, there is still no definite evidence from the experimental and theoretical results. In addition, the question arises whether replacing some of the Nb^5+^ with other pentavalent ions such as Ta^5+^ can affect the dielectric and electrical properties of CaCu_3_Ti_4-*x*_(Nb_1/2_Al_1/2_)*_x_*O_12_ ceramics. How do the charge-compensation mechanisms occurring in the CaCu_3_Ti_4-*x*_(Nb_1/2_Al_1/2_)*_x_*O_12_, in which Nb^5+^ was partially replaced by Ta^5+^, differ from CaCu_3_Ti_4-*x*_(Nb_1/2_Al_1/2_)*_x_*O_12_ These questions should be clearly carried out. Furthermore, to achieve the colossal permittivity in codoped TiO_2_−, NiO−, and SnO_2_−based oxides, these oxides must be sintered at high temperatures (1200–1450 °C) [1,15,37]. Thus, this work aims to theoretically and experimentally describe the effects of charge-compensation mechanisms and associated formation of defects on the microstructure evolution, colossal dielectric permittivity, and electrical properties of Ta^5+^-doped CaCu_3_Ti_4-*x*_(Nb_1/2_Al_1/2_)*_x_*O_12_ ceramics, which were fabricated at a relatively low temperature.

In this work, CaCu_3_Ti_4-*x*_(Ta_0.25_Nb_0.25_Al_0.5_)*_x_*O_12_ ceramics with *x* = 0, 0.025, and 0.05 were prepared by a solid-state reaction method and sintered at 1090 °C for 18 h. Significantly enhanced dielectric properties of CCTO were obtained by doping with acceptor-Al^3+^ and donor-Nb^5+^/Ta^5+^. The first principle calculations were performed to predict the preferential sites of dopants in the CCTO structure and, hence, defect structures. Largely improved colossal dielectric properties of CaCu_3_Ti_4-*x*_(Ta_0.25_Nb_0.25_Al_0.5_)*_x_*O_12_ ceramics were described based on the theoretical and experimental results. The possible mechanisms of charge compensation were discussed based on the theoretical calculation.

## 2. Experimental Details

Nb_2_O_5_ (Sigma-Aldrich, 99.99%), Ta_2_O_5_ (Aldrich, 99.99% purity), Al_2_O_3_ (Sigma-Aldrich, 99.99% purity), CaCO_3_ (Sigma-Aldrich, 99.0% purity), CuO (Merck, 99.9% purity), and TiO_2_ (Sigma-Aldrich, 99.99% purity) are the starting raw materials for the preparation of CaCu_3_Ti_4-*x*_(Ta_0.25_Nb_0.25_Al_0.5_)*_x_*O_12_ ceramics. Details of the preparation method were provided in our previous publication [2]. The CaCu_3_Ti_4-*x*_(Ta_0.25_Nb_0.25_Al_0.5_)*_x_*O_12_ ceramic samples were obtained by sintering at 1090 °C for 18 h. The CaCu_3_Ti_4-*x*_(Ta_0.25_Nb_0.25_Al_0.5_)*_x_*O_12_ samples with *x* = 0, 0.025, and 0.05 are referred to as the CCTO, NbTaAl025, and NbTaAl05 ceramics, respectively.

The crystalline structure of samples was characterized by X-ray Diffractometer (PANalytical model EMPYREAN). The X-ray diffraction (XRD) data were collected in the 2θ range of 20–80° by using step increases of 0.02°/point. Rietveld quantitative phase analysis was carried out using the X’Pert High Score Plus v3.0e software package by PANalytical. The parameters and coefficients optimized were the zero shift, scale factor, background (with function type: polynomial), profile half-width parameters (v, u, w), lattice parameters (a, b, c), atomic site occupancies (Wyckoff), and preferred orientation parameter. Surface morphologies of samples were studied using Desktop Scanning Electron Microscopes (SEC, SNE-4500M).

The polished samples were coated by the conductive silver paint and heated in a furnace at 600 °C for 30 min using a heating rate of 5 °C/min. The dielectric properties of CaCu_3_Ti_4-*x*_(Ta_0.25_Nb_0.25_Al_0.5_)*_x_*O_12_ ceramics were measured by a KEYSIGHT E4990A Impedance Analyzer with an oscillation voltage of 500 mV in the frequency range of 40–10^7^ Hz. The temperature dependence of the dielectric properties was performed in the temperature range from −60 to 210 °C. Nonlinear current density-electric field (*J*–*E*) characteristics were measured using a high voltage measurement unit (Keithley model 247). The electric field breakdown (*E*_b_) was achieved at *J* =1 mA.cm^−2^. The nonlinear coefficient (*α*) was calculated in the range of J = 1−10 mA.cm^−2^.

The preferential configuration for the Nb, Ta, and Al occupying in the Ti sites of the CCTO structure was predicted by DFT calculations. The unit cell of the CCTO structure with 40 atoms was used. Details of the calculation method are reported in our previous publication [38]. For Ta or Nb, the 5*p*, 6s, and 5*d* states were used. The 3*s* and 3*p* valence states were chosen for the Al pseudopotential. 

## 3. Results and Discussion

**Figure 1:**
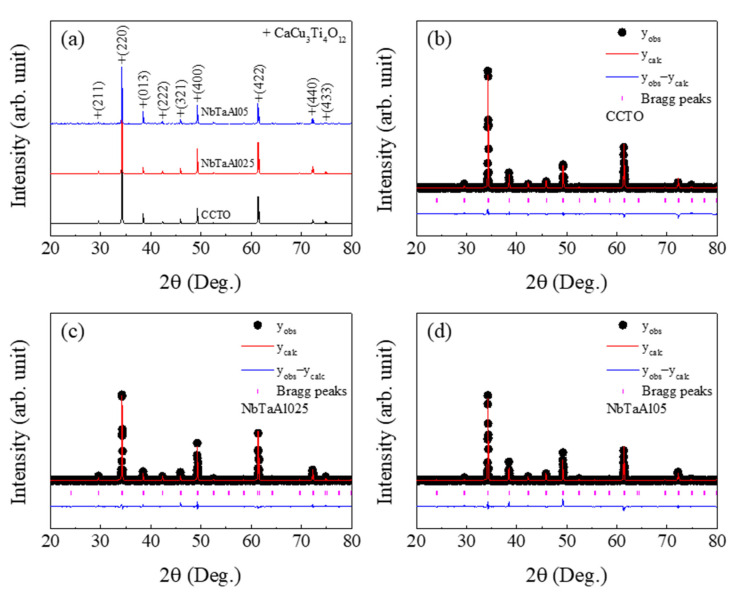
(**a**) XRD patterns of the CCTO, NbTaAl025, and NbTaAl05 ceramics. (**b**–**d**) The Rietveld profile fits of the CCTO, NbTaAl025, and NbTaAl05 ceramics, respectively.

Identification of the crystalline structure of samples was studied using an XRD technique, as shown in Figure 1a.

A single phase of CCTO can be detected in all samples. No impurity phase related to dopant elements, e.g., Ta, Nb, and Al, is observed. As illustrated in Figure 1b–d, all the XRD patterns can be well fitted by the Rietveld method. Structural data obtained from the Rietveld refinement were given in Table 1. *R*_exp_ (expected), *R*_p_ (profile), and *R*_wt_ (weighted profile) of the CCTO, NbTaAl025, and NbTaAl05 were lower than 10, while the factor of the goodness of fit (GOF) of all samples was very low (1 < GOF < 2). Lattice parameter (*a*) of all the samples were nearly in a value of 7.391 Å for the standard CCTO structure [12]. The *a* values of the CCTO, NbTaAl025, and NbTaAl05 were 7.394, 7.393, and 7.393 Å, respectively. The *a* values of both the NbTaAl025 and NbTaAl05 ceramics were slightly less than that value of the CCTO ceramic. The *a* values of the NbTaAl025 and NbTaAl05 ceramics are equal to the *a* values of the CaCu_3_Ti_4-*x*_(Nb_0.5_Al_0.5_)*_x_*O_12_ ceramics with *x* = 0.025 and 0.05, respectively [2].

A slight change in the *ɑ* values of the NbTaAl025, and NbTaAl05 compared to that of the CCTO are associated with a slight difference between the average ionic radii of all the dopants of $r_{average}=0.588\;\dot{A}$ (i.e., $r_{Al^{3+}}=0.535\;\dot{A}$, $r_{Nb^{5+}}=0.640\;\dot{A}$, and $r_{Ta^{5+}}=0.640\;\dot{A}$) and the host Ti^4+^ ($r_{Ti^{4+}}=0.605\;\dot{A}$) ion [39]. According to this result, it is likely that the dopants could completely substitute into the CCTO structure. 

**Figure 2:**
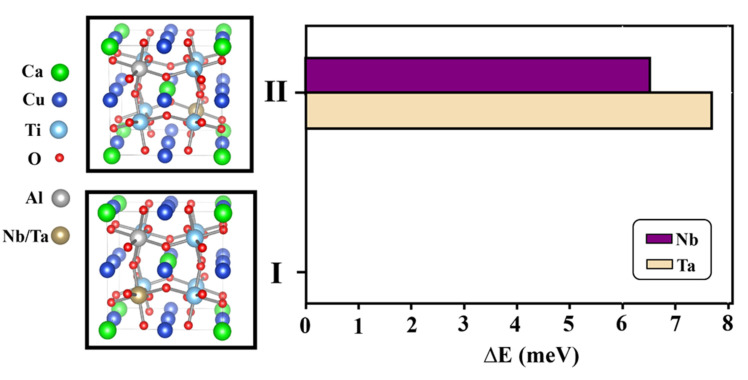
Total energy of Nb/Ta and Al codoped CCTO structure; structure-I shows Nb/Ta and Al atoms are close to each other and structure-II shows Al atom is far from Nb/Ta atom.

To further confirm the assumption for the substitution of the dopants, preferential site occupancy of the dopants and arrangement of the dopants in the structure were calculated using the DFT technique. As demonstrated in Figure 2, two different initial-defect configurations are designed in the Ti^4+^ sites. For both structure-I and structure-II, Nb/Ta and Al are in the octahedral sites of Ti. The positions of Nb/Ta and Al in the octahedral sites of structure-II are apart. On the other hand, the positions of Nb/Ta and Al in the octahedral sites of structure-I are closed together. According to the calculated total energies for these two structures, it can be confirmed that the Al atom is close to Nb/Ta atom in the CCTO structure (structure-I). This is because the total energy of structure-II is higher than that of structure-I. For Al and Nb in octahedral sites, the total energy difference between these two structures is 6.52 meV. Moreover, the total energy difference is 7.68 meV for the case of Al and Ta in octahedral sites. The adjacent Al-Nb and Al-Ta prefer to form in the CCTO structure.

Charge compensation is usually required for doping CCTO with Nb^5+^/Ta^5+^ into Ti^4+^ sites, giving rise to the reduction of Ti^4+^ to Ti^3+^ (${\mathrm{Ti}}^{4+}+e^{-}\to {\mathrm{Ti}}^{3+}$) as an equation,

(1) $${\mathrm{Nb}}_{2}O_{5}/{\mathrm{Ta}}_{2}O_{5}+2{\mathrm{TiO}}_{2}\overset{4{\mathrm{TiO}}_{2}}{\to }2{\mathrm{Ti}}_{\mathrm{Ti}}^{\prime }+2{\mathrm{Nb}}_{\mathrm{Ti}}^{•}/2{\mathrm{Ta}}_{\mathrm{Ti}}^{•}+8O_{O}+1/2O_{2}$$

Charge compensation is also required for doping CCTO with Al^3+^ into Ti^4+^ sites by the creation of an oxygen vacancy ($V_{O}^{••}$), as illustrated by equation,

(2) $${\mathrm{Al}}_{2}O_{3}\overset{2{\mathrm{TiO}}_{2}}{\to }2{\mathrm{Al}}_{\mathrm{Ti}}^{\prime }+V_{O}^{••}+3V_{O}$$

Equations (1) and (2) can be applied in the case of structure-II or in the cases of single-doped CCTO ceramics with Nb^5+^/Ta^5+^ or Al^3+^ into Ti4+ sites. 

According to the DFT calculations, Al and Nb/Ta in each octahedral site prefer to close together, as demonstrated in structure-I. Charge compensation is not required due to self-charge compensation, following relation:

(3) $${\mathrm{Nb}}_{2}O_{5}/{\mathrm{Ta}}_{2}O_{5}+{\mathrm{Al}}_{2}O_{3}\overset{4{\mathrm{TiO}}_{2}}{\to }2{\mathrm{Al}}_{\mathrm{Ti}}^{\prime }+2{\mathrm{Nb}}_{\mathrm{Ti}}^{•}/2{\mathrm{Ta}}_{\mathrm{Ti}}^{•}+8V_{O}$$

The self-charge compensation mechanism may have a remarkable effect on the electrical properties of the grains and grain boundaries, resulting in the dielectric and non-Ohmic properties of the NbTaAl025 and NbTaAl05.

**Figure 3:**
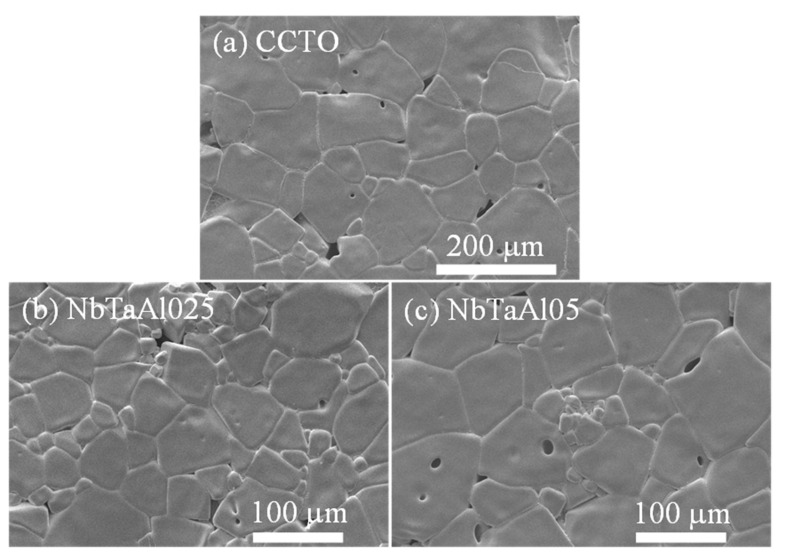
Secondary electron images with magnification of 200× of surface morphologies of (**a**) CCTO, (**b**) NbTaAl025, and (**c**) NbTaAl05 ceramics using scanning electron microscope with a W filament as an electron source.

The SEM images of the surface microstructures of the CCTO, NbTaAl025, and NbTaAl05 are shown in Figure 3a–c. The mean grain sizes of all samples were summarized in Table 1. The mean grain sizes of the CCTO, NbTaAl025, and NbTaAl05 were 96.5 ± 25.8, 61.2 ± 13.6, and 80.7 ± 22.4 μm, respectively. The mean grain sizes of the NbTaAl025 and NbTaAl05 are smaller than that of the CCTO. According to our previous works [2,19], the grain sizes of Nb^5+^ and Ta^5+^ single-doped CCTO ceramics were reduced compared to that of the undoped CCTO. On the other hand, the grain size of CCTO ceramics was considerably enlarged by the addition of Al^3+^ due to the dominant effect of the oxygen vacancy diffusion (or relatively related diffusion of oxygen ions) [2]. The grain growth of polycrystalline ceramics is driven by the grain boundary mobility, which can be enhanced by increasing the diffusion rate of ions or charged species across the grain boundary. The mean grain size of the NbTaAl025 and NbTaAl05 cannot be increased compared to that of the CCTO due to the self-charge compensation mechanism (Equation (3)). Furthermore, it is possible that the substitution of Nb^5+^/Ta^5+^ ions may also be ionically compensated by cation vacancies ($V_{Ti}^{⁗}$). Trapped $V_{Ti}^{⁗}$ in the negative space-charge region is likely related to a depletion of the intrinsic defect of oxygen vacancies in the space-charge region [40]. The diffusion rate of oxygen ions across the grain boundary is therefore reduced owing to the sizeable ionic size of oxygen ions.

**Figure 4:**
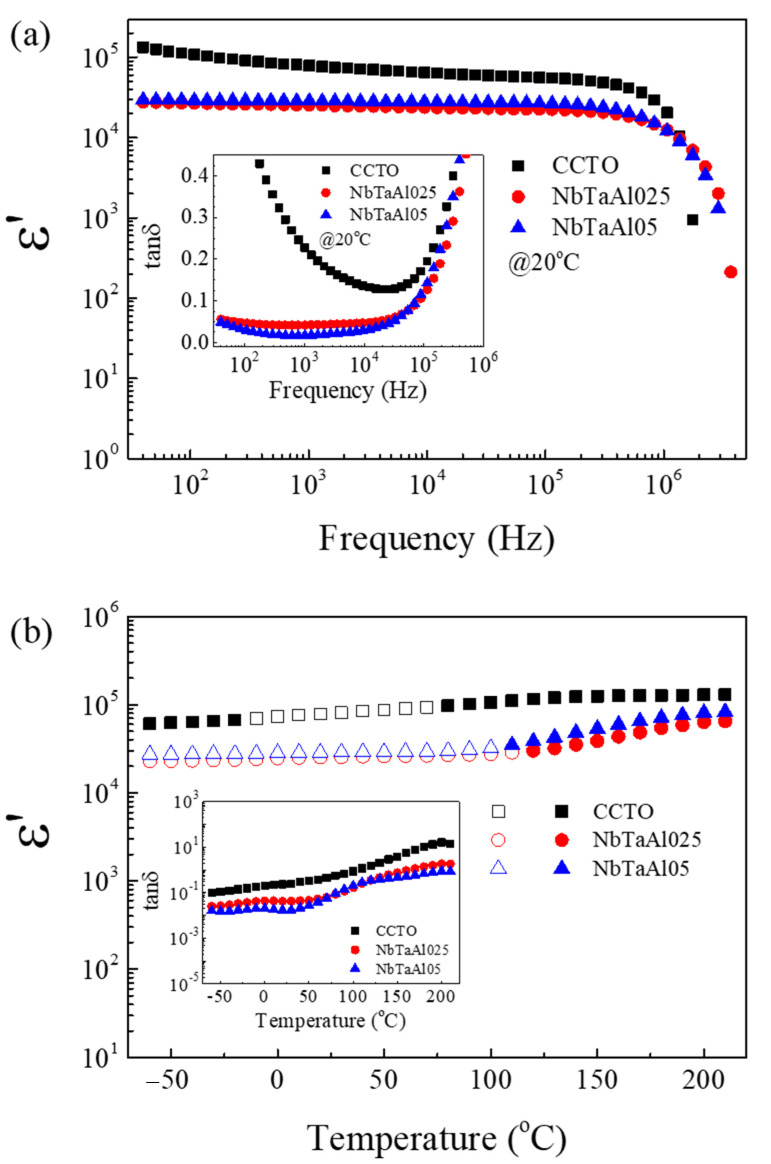
(**a**). Frequency dependence of *ε*′ at 20 °C for CCTO, NbTaAl025, and NbTaAl05 ceramics; its inset shows frequency dependence of tanδ. (**b**) Temperature dependence of *ε*′ at 1 kHz for CCTO, NbTaAl025, and NbTaAl05 ceramics; its inset shows temperature dependence of tanδ at the same frequency.

The frequency dependence of *ε*′ and tan*δ* for the CCTO, NbTaAl025, and NbTaAl05 are shown in Figure 4a.

A low-frequency *ε*′, which was contributed from grain boundary response of these ceramics, was slightly dependent with frequency in the range of ~40 to 4 × 10^6^ Hz. The rapid decrease in *ε*′ at 20 °C appeared in a frequency range of >10^6^ Hz, corresponding to the dramatically increased tanδ in a high-frequency range, as shown in its inset of Figure 4a. This dielectric behavior is referred to as the dielectric relaxation behavior, which is usually observed in CCTO ceramics [28,41,42,43]. The values of *ε*′ and tanδ at 1 kHz and 20 °C of all the samples were summarized in Table 2. 

Clearly, the colossal dielectric properties were observed in all the samples. According to the SEM images in Figure 2, it can be suggested that the close relationship between the mean grain sizes and the low-frequency *ε*′ that giant dielectric response of CCTO is associated with the IBLC model [41]. Accordingly, the colossal permittivity can be expressed as

ε′ = ε_gb_*G*/*t*_gb_ (4)

where *G* is the mean grain size, ε_gb_ and *t*_gb_ are the dielectric permittivity and thickness of the GB, respectively. Thus, variation in the ε′ values should be correlated to the changes in *G*.

Interestingly, the low-frequency tan*δ* of CCTO was significantly decreased by doping with Al^3+^ and Nb^5+^/Ta^5+^, as demonstrated in an inset of Figure 4a (for CaCu_3_Ti_4-*x*_(Ta_0.25_Nb_0.25_Al_0.5_)*_x_*O_12_). The tan*δ* values at 1 kHz of the CCTO, NbTaAl025, and NbTaAl05 ceramics were 0.227, 0.042, and 0.017, respectively. Notably, tan*δ* at 1 kHz of the NbTaAl05 (*x* = 0.05) was reduced by a factor of 13 compared to that of the CCTO, while, at 40 Hz, tan*δ* was reduced by a factor of 15. Moreover, the tan*δ* values of the NbTaAl025 and NbTaAl05 were lower than 0.1 over the frequency range of 40−10^5^ Hz. Strongly decreased tanδ of the doped samples may be attributed to the enhanced grain boundary properties as a result of Nb^5+^/Ta^5+^ and Al^3+^. According to our previous publication [2], high *ε*′ with low tan*δ* can be obtained in the CaCu_3_Ti_4-*x*_(Nb_1/2_Al_1/2_)*_x_*O_12_. It can be confirmed that by partially replacing Nb^5+^ with Ta^5+^, the improved colossal dielectric properties can be achieved. Furthermore, the tan*δ* can be further reduced by doping Ta5+ into CaCu_3_Ti_4-*x*_(Nb_1/2_Al_1/2_)*_x_*O_12_.

The temperature dependence of the colossal dielectric properties (*ε*′ and tanδ) at 1 kHz is revealed in Figure 4b and its inset. Open symbols signify the variation of *ε*′ in each sample of ≤±15% compared to its value at ~25 °C. The temperature stability of *ε*′ was improved by doping with Nb^5+^, Ta^5+^, and Al^3+^. The increase in *ε*′ in a high-temperature range is usually observed in CCTO-based ceramics [19,20,28,29,30,31,32]. As shown in the inset, the tan*δ* of the NbTaAl025 and NbTaAl05 were lower than that of the CCTO ceramic in over the temperature range of −60 to 210 °C. Enhancement of colossal dielectric properties as well as the temperature stability of *ε*′ in the NbTaAl025 and NbTaAl05 may be correlated with the enhancement of the electrical response at the grain boundaries.

**Figure 5:**
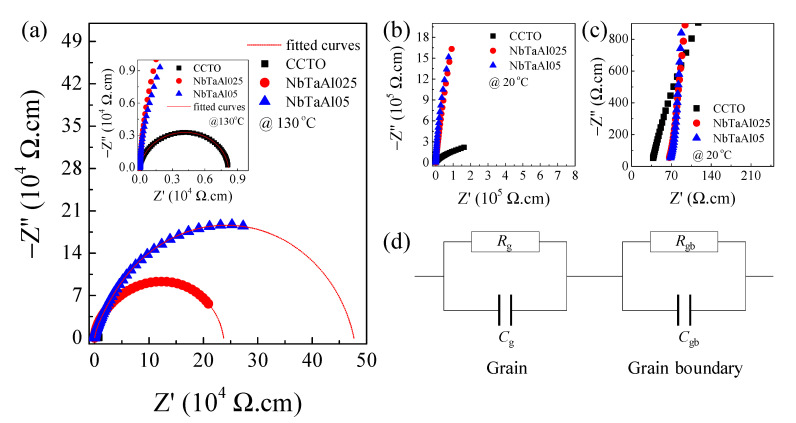
(**a**). Impedance complex Z* plots at 130 °C of the CCTO, NbTaAl025, and NbTaAl05 ceramics; its inset shows the enlarged scale of Z* plots at 130 °C. (**b**,**c**) the low- and high-frequency of Z* plot at 20 °C of these samples, respectively. (**d**) Equivalent circuit represented the electrical heterogeneous microstructure of semiconducting grain and insulating grain boundary.

To clarify the enhanced colossal dielectric properties of the NbTaAl025 and NbTaAl05, an impedance spectroscopy technique was used to study the effects of dopants on the electrical properties of the grains and grain boundaries. To analyze the impedance data, Z* plots were modelled by an ideal equivalent circuit of two parallel *RC* elements. The first *RC* element for the grain and the second element for the grain boundary response are connected in series. The complex impedance (Z*) can be calculated using the equation,

(5) $$Z^{*}=Z^{\prime }-iZ^{\prime \prime }=\frac{1}{i\omega C_{0}\left(\varepsilon^{\prime }-i\varepsilon^{\prime \prime }\right)}$$

where ε* = *ε*′ − *iε*″ is the complex dielectric permittivity, *ω* = 2π*f* is the angular frequency, and *C*_0_ = *ε*_0_*S*/*t* is the capacitance of free space. *S* and *t* are the electrode area and sample thickness, respectively. The grain resistance (*R*_g_) can be estimated from the nonzero intercept at high frequencies of the Z* plane plot, while the grain boundary resistance (*R*_gb_) can be estimated from the diameter of a large semicircle arc [27,44]. The semicircular arcs of the NbTaAl025 and NbTaAl05 cannot be observed at a low temperature. As a result, the variation in *R*_gb_ values cannot be observed. Thus, the Z* plots at 130 °C of the CCTO, NbTaAl025, and NbTaAl05 are represented, shown in Figure 5a and its inset. It can be shown that *R*_gb_ of CCTO significantly increased with increasing dopant content. As expected, the great decrease in tan*δ* was due to the significantly increased *R*_gb_ values. The lowest *R*_gb_ was observed in the CCTO ceramic, as demonstrated in the inset. The *R*_gb_ values at 130 °C of the CCTO, NbTaAl025, and NbTaAl05 ceramics at 130 °C were 6.52 × 10^3^, 1.86 × 10^5^, and 3.74 × 10^5^ Ω·cm, respectively. The enhanced *R*_gb_ of CCTO can be created by doping with Nb^5+^, Ta^5+^, and Al^3+^.

As illustrated in Figure 5b, although the *R*_gb_ value cannot be calculated in the Z* plots at 20 °C, it can be reasonably indicated that *R*_gb_ at around room temperature of the NbTaAl025 and NbTaAl05 was much larger than that of the CCTO. Therefore, at 20 °C, a significantly reduced tan*δ* in the NbTaAl025 and NbTaAl05 was resulted from their vastly enhanced *R*_gb_ values. The close relationship between the significant increase in *R*_gb_ and a reduced tanδ is similar to those report in literature [2,17,30,31,32]. Doping CCTO with only Nb^5+^ or Ta^5+^ resulted in a significant reduction in the *R*_gb_ compared to that of the undoped CCTO [2,18,19]. However, in this current study, the addition of small Al^3+^ content can recover a sizeable *R*_gb_ value, which was larger than that of the undoped CCTO by two orders of magnitude. Figure 5c shows the Z* plots at high frequencies and 20 °C, showing the nonzero intercept on the Z′ axis. Accordingly, the *R*_g_ values at 20 °C of all the samples can be obtained and found to be 35, 63, and 65 Ω.cm for the CCTO, NbTaAl025, and NbTaAl05, respectively. According to Equation (1), *R*_g_ of the NbTaAl025 and NbTaAl05 should be decreased due to the introduction of free electrons. Conversely, *R*_g_ of the NbTaAl025 and NbTaAl05 increased slightly. Therefore, the effect of self-charge compensation, as predicted by the theoretical calculation, was dominant. Furthermore, few portions of $V_{Ti}^{⁗}$ may be induced because the substitution of Nb^5+^/Ta^5+^ into CCTO could be ionically compensated by the creation of $V_{Ti}^{⁗}.$

**Figure 6:**
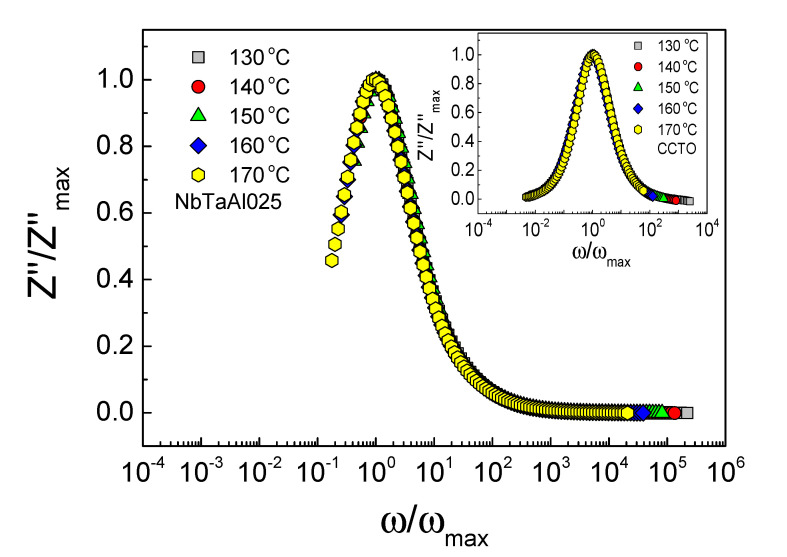
Scaling behavior of Z″ of the NbTaAl05 ceramic in the temperature range from 130 to 170 °C; its inset shows scaling behavior of Z″ of the CCTO sample.

The scaling behavior of *Z*″ in the temperature range of 130–2212170 °C was investigated, as shown in Figure 6 and the inset. The perfect overlap of all curves at different temperatures into a single curve is observed for the NbTaAl05 and CCTO ceramics. This result confirmed that the relaxation process was originated from the same mechanism.

**Figure 7:**
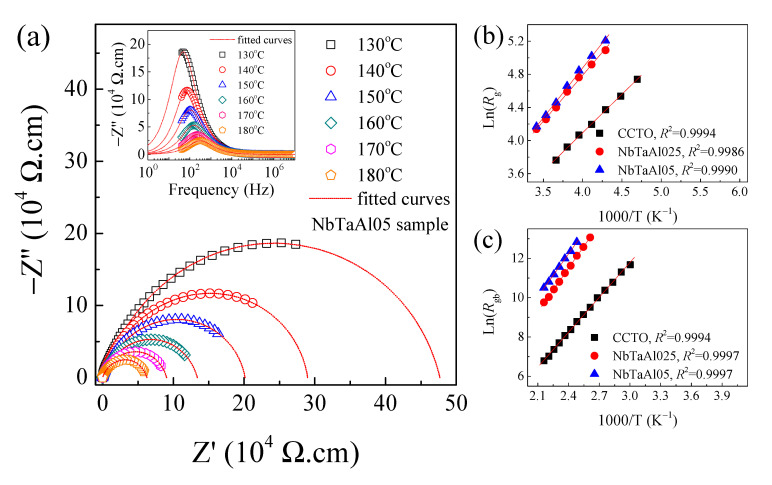
(**a**). Impedance complex Z* plots in a temperature range of 130–180 °C of the NbTaAl05 ceramic; its inset shows the frequency dependence of Z″ in the same temperature range. (**b**) Arrhenius plot of *R*_g_ and (**c**) Arrhenius plot of *R*_gb_.

To understand the electrical properties of the grain boundary, Z* plots at different temperatures were studied. Figure 7a and its inset show semicircle arcs of Z* plots and the frequency dependence of *Z*″ in the temperature range of 130–180 °C.

The diameter of the arc increased with decreasing temperature, indicating the increased *R*_gb_ value. Furthermore, the *Z*″_max_ value also increased as the temperature decreased. *R*_g_ was also increased with increasing temperature (not show). The *R*_g_ and *R*_gb_ values at different temperatures can be obtained. The variations of *R*_g_ and *R*_gb_ with temperature follow the Arrhenius law [2,19,27],

(6) $$R_{g,\mathrm{gb}}=R_{0}\exp\left(\frac{E_{g,\;\mathrm{gb}}}{k_{B}T}\right)$$

where *R*_0_ is a pre-exponential constant term. *k*_B_ and *T* are the Boltzmann constant and absolute temperature, respectively. *E*_g_ and *E*_gb_ are the conduction activation energies of the grains and grain boundaries, respectively. As demonstrated in Figure 7b,c, the temperature dependences of *R*_g_ and *R*_gb_ are well fitted by the Arrhenius law, Equation (6). The *E*_g_ and *E*_gb_ can be calculated from the slopes of the fitting lines. The *E*_g_ and *E*_gb_ of all samples are summarized in Table 2. The *E*_g_ values of the CCTO, NbTaAl025, and NbTaAl05 were 0.080, 0.096, and 0.104 eV, respectively. *E*_g_ slightly changed as the doping content was different, corresponding to a slight increase in *R*_g_. The *E*_gb_ values were 0.510, 0.641, and 0.627 eV, respectively. Interestingly, Doping CCTO with Nb^5+^/Ta^5+^ and Al^3+^ can cause an increase in the *E*_gb_. According to the SEM images, the mean grain sizes of the NbTaAl025 and NbTaAl05 were smaller than that of the undoped CCTO. Thus, the density of the insulating grain boundaries in the NbTaAl025 and NbTaAl05 is higher than that of the CCTO. This is the first reason for the enhanced *R*_gb_ values of the NbTaAl025 and NbTaAl05. Another reason is the increase in *E*_gb_, which indicated the enhanced Schottky barrier at the grain boundaries (Ф_b_). These are possible mechanisms on the enhanced dielectric properties in the NbTaAl025 and NbTaAl05 [2,30,31,32]. According to the IBLC model of the Schottky barrier at the grain boundaries [44], Ф_b_ is inversely proportional to the charge carrier concentration inside the semiconducting grains (*N*_d_) or proportional to *R*_g_. As shown in Figure 5c, a slight increase in *R*_g_ of the NbTaAl025 and NbTaAl05 was the primary cause of the increased Ф_b_.

**Figure 8:**
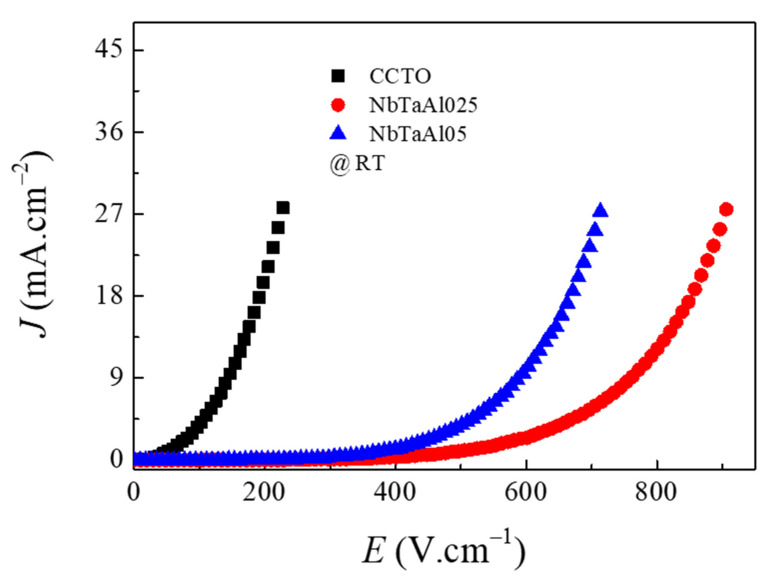
Nonlinear *J*–*E* characteristics of the CCTO, NbTaAl025, and NbTaAl05 ceramics at room temperature.

The nonlinear *J*–*E* properties are shown in Figure 8. All the samples can exhibit the non-Ohmic characteristics of the *J*–*E* curves. The breakdown electric field (*E*_b_) and nonlinear coefficient (*α*) of samples were summarized in Table 2. The *α* values of the CCTO, NbTaAl025, and NbTaAl05 were 2.13, 5.21, and 5.02, respectively, while their *E*_b_ values were 52, 499, and 381 V·cm^−1^, respectively. The nonlinear *J*–*E* properties of CCTO were improved by doping with Nb^5+^/Ta^5+^ and Al^3+^. Although *R*_gb_ of the NbTaAl05 ceramic was larger than *R*_gb_ of the NbTaAl025 ceramic, *E*_b_ of the NbTaAl05 ceramic was smaller than that of NbTaAl025 ceramic. This may be due to the effect of grain size on the NbTaAl05 was more significant than the size of NbTaAl025. The nonlinear *J*–*E* properties of CCTO-based oxides were originated from the formation of the Schottky barrier at the grain boundaries. According to previous works [19,45], the non-Ohmic properties of CCTO were reduced by doping with Ta^5+^ and Nb^5+^. It was suggested that the negative charge of unknown acceptors was compensated by the positive charge of Nb^5+^/Ta^5+^, resulting in a decrease in the number of active acceptors, which lead to the existence of Ф_b_. In this current study, the positive charges of Nb^5+^/Ta^5+^ were compensated by the effectively negative charge of $2Al_{Ti}^{\prime }$ (Equation (3)) inside the grains without any effect on the number of active acceptors. Thus, the Ф_b_ of the NbTaAl025 and NbTaAl05 cannot be reduced by the introduction of Nb^5+^/Ta^5+^.

**Figure 9:**
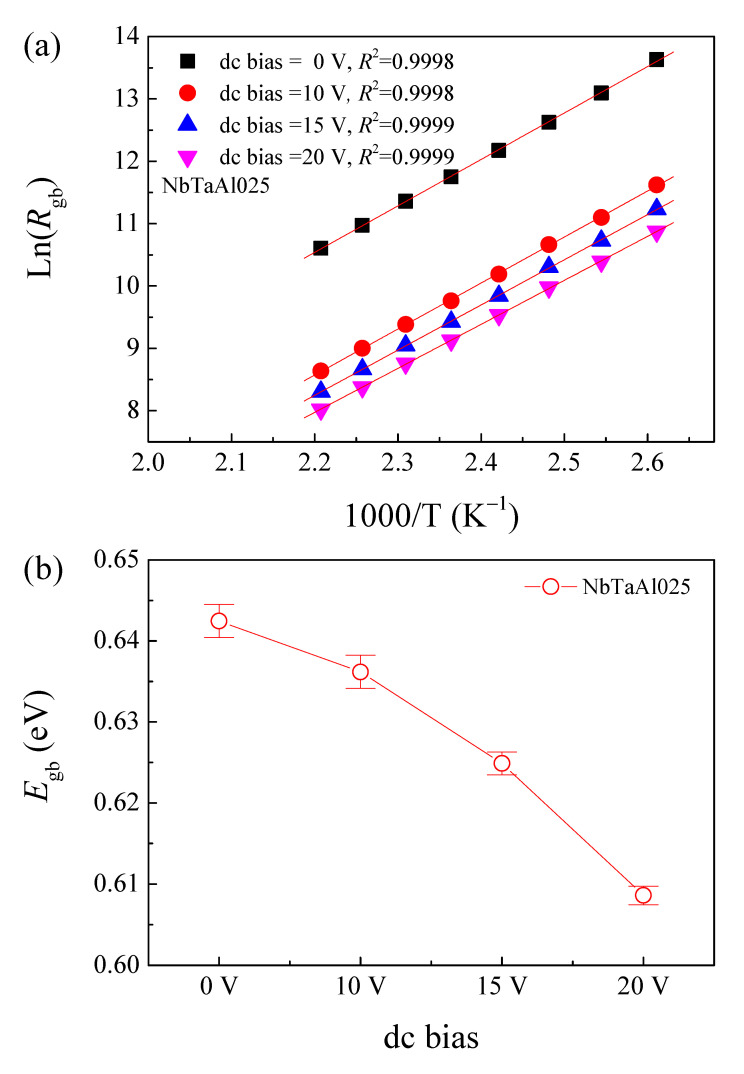
(**a**). Arrhenius plot of *R*_gb_ for the selected NbTaAl025 sample under the dc bias range of 0–20 V. (**b**) Correlation between dc bias and *E*_gb_ of this sample.

Characterization of the electrical response of the grain boundaries was investigated under DC bias. As illustrated in Figure 9a, *R*_gb_ of the NbTaAl025 decreased with increasing DC bias, indicating a decrease in Ф_b_ in the forward direction. At different DC bias levels, the temperature dependence of *R*_gb_ for the NbTaAl025 ceramic was well fitted by the Arrhenius law. As demonstrated in Figure 9b, *E*_gb_ of the NbTaAl025 ceramic decreased with increasing DC bias. The *E*_gb_ values at 0, 10, 15, and 20 DC bias voltage were 0.642 ± 0.002, 0.636 ± 0.002, 0.625 ± 0.001, and 0.609 ± 0.001 eV, respectively. As can be seen in Figure 9b, the error bars are too small, indicating the significant decrease in the *E*_gb_ values as a result of DC bias. Clearly, the DC bias reduced the *E*_gb_, indicating a decrease of the potential barrier height at the grain boundaries. This result is very consistent with the results in the research work of Adams, et al. [44]. The DC bias experiment shows the close correlation between the grain boundary response and Schottky barrier response. Thus, the colossal permittivity in the CCTO-based oxides can be described by the IBLC model of the Schottky barrier at the internal insulating layer. In this model, all the grains (or grain boundaries) must show similar properties. So that all grains (or all grain boundaries) can be averaged by a single equivalent circuit component. The deviation from in the linear relationship of the *E*_gb_ and DC bias voltage may be due to the different microstructures between the ideal microstructure and the fabricated microstructure of the sintered ceramics.

## 4. Conclusions

CaCu_3_Ti_4-*x*_(Al_1/2_Ta_1/4_Nb_1/4_)*_x_*O_12_ (*x* = 0−0.05) ceramics with a single-phase of CCTO were synthesized by a solid-state reaction method. The Nb^5+^/Ta^5+^ and Al^3+^ dopants had no effect on the crystal structure, while the microstructure evolution of CCTO ceramics was remarkably resulted by the dopants. Interesting, tan*δ* was decreased by a factor of 13 (tanδ ~0.017), while the colossal permittivity of ε′ ~ 10^4^ was achieved. The great increase in *R*_gb_ by two orders of magnitude was the primary cause for the observed decrease in tanδ. The first principle calculations confirmed that Al and Nb/Ta were closed together in the octahedral Ti sites, leading to self-charge compensation. Ф_b_ of CCTO ceramics can be increased by doping with Nb^5+^/Ta^5+^ and Al^3+^ due to the self-charge compensation and the decrease in *R*_g_. Therefore, the improved colossal dielectric properties of the NbTaAl025 and NbTaAl05 were attributed to the enhanced electrical properties of the grain boundaries. The substitution of Nb^5+^/Ta^5+^ and Al^3+^ into CCTO ceramics can cause improved non-Ohmic properties, which were originated by the increased Ф_b_ and increased grain boundary density. Ф_b_ was reduced by applying DC bias, indicating the formation of the Schottky barrier type. The colossal dielectric properties and non-Ohmic characteristics can be well explained based on the formation of the Schottky barrier in the IBLC structure.

## Figures and Tables

**Table 1:** Structural data obtained from the Rietveld refinement and mean grain size (*G*) for CCTO, NbTaAl025, and NbTaAl05 ceramics.

Sample	CCTO	NbTaAl025	NbTaAl05
*a* (Å)	7.394	7.393	7.393
*R*_exp_ (%)	5.323	5.309	5.406
*R*_p_ (%)	4.200	3.758	4.473
*R*_wt_ (%)	6.703	5.601	7.355
GOF	1.586	1.113	1.851
*G* (μm)	96.5 ± 25.8	61.2 ± 13.6	80.7 ± 22.4

**Table 2:** *ε*′ at 10^3^ Hz and 20 °C, *R*_g_ at 20 °C, *R*_gb_ at 130 °C, Activation energies of grains (*E*_g_) and GBs (*E*_gb_), Breakdown electric field (*E*_b_), and Nonlinear coefficient (*α*) of the CCTO, NbTaAl025, and NbTaAl05 ceramics.

Sample	*ε*′	tanδ	*R*_g_ (Ω.cm)	*R*_gb_ (Ω.cm)	*E*_g_ (eV)	*E*_gb_ (eV)	*E*_b_ (V/cm)	*α*
CCTO	7.87 × 10^4^	0.227	35	6.52 × 10^3^	0.080	0.510	52	2.13
NbTaAl025	2.52 × 10^4^	0.042	63	1.86 × 10^5^	0.096	0.655	499	5.21
NbTaAl05	2.86 × 10^4^	0.017	65	3.74 × 10^5^	0.104	0.634	381	5.02

## Data Availability

The data presented in this study are available in article.
